# Polymicrobial bacteremia after treatment of transcatheter arterial chemoembolization: A case report

**DOI:** 10.1097/MD.0000000000017393

**Published:** 2019-10-04

**Authors:** Xue-Yao Fang, Long-Hua Hu, Ya-Ping Hang, Yan-Hui Chen, Xiu-Hua Xu, Xiao-Yan Hu, Yan-Hua Liu, Nan Zhang, Qiao-Shi Zhong

**Affiliations:** Department of Clinical Laboratory Medicine, the Second Affiliated Hospital of Nanchang University, Key Laboratory of Laboratory Medicine in Jiangxi Province, China.

**Keywords:** *Aeromonas hydrophila*, *Clostridium perfringens*, liver abscess, polymicrobial bacteremia, TACE

## Abstract

**Rationale:**

Bacteremia caused by polymicrobial infections are rare but dangerous. We report a case of hepatic abscess combined with polymicrobial bacteremia in a 49-year-old male patient after surgery and transcatheter arterial chemoembolization (TACE).

**Patient concerns:**

The patient was admitted to hospital with metastatic liver cancer for periodic chemotherapy and developed a high fever and tenderness to the liver following surgery and TACE.

**Diagnosis:**

Hepatic abscess combined with polymicrobial bacteremia.

**Interventions:**

The clinician formulated a therapy in accordance with the drug susceptibility test and the empirical drug use for anaerobic bacteria. A comprehensive treatment plan was adopted, on the basis of the combination of nitrazole and imipenem as anti-infection drugs as well as continuous abscess drainage.

**Outcomes:**

After comprehensive therapy, the patient was ultimately discharged without any residual symptoms.

**Lessons:**

Bloodstream infection caused by multiple bacteria increases the difficulty of anti-infection treatments, leading to poor treatment outcome and high mortality. Therefore, a fast and accurate diagnosis of polymicrobial bacteremia is key for initiation of an effective antimicrobial treatment. Additionally, pre-operative prophylactic antibiotics are advisable when patients have a history of abdominal surgery and are immune-compromised.

## 1 Introduction

*Clostridium perfringens* and *Aeromonas hydrophila* can be isolated from human gastrointestinal and intra-abdominal sites and can lead to both gastrointestinal and non-gastrointestinal infections under certain circumstances.^[1,2]^ Among these infections, sepsis is a rare phenomenon, with its clinical manifestations being unexplained chills, high fever and abdominal pain. Inappropriate treatment can be fatal. *Escherichia coli* is a common conditional pathogen in the clinic and a major origin of bacterial bloodstream infection (BSI). Underlying the disease, long-term hospitalization, invasive surgery and intensive-care units are all risk factors for patients susceptible to BSI caused by *E coli*.^[3]^

BSI caused by multiple bacteria increases the difficulty of anti-infection treatments, leading to a poor treatment outcome and high mortality. Therefore, a fast and accurate identification of polymicrobial bacteremia is key to initiate an effective antimicrobial coverage. Whether patients need prophylactic antibiotic administration to reduce post-operative infections before TACE remains unclear.^[4]^ Pre-operative prophylactic antibiotics are necessary when a patient has a history of abdominal surgery and is immune compromised.

This case report was approved by the ethics committee of the Second Affiliated Hospital of Nanchang University, Nanchang, China, and informed consent form was given by the patient.

## 2 Case report

**Figure 1:**
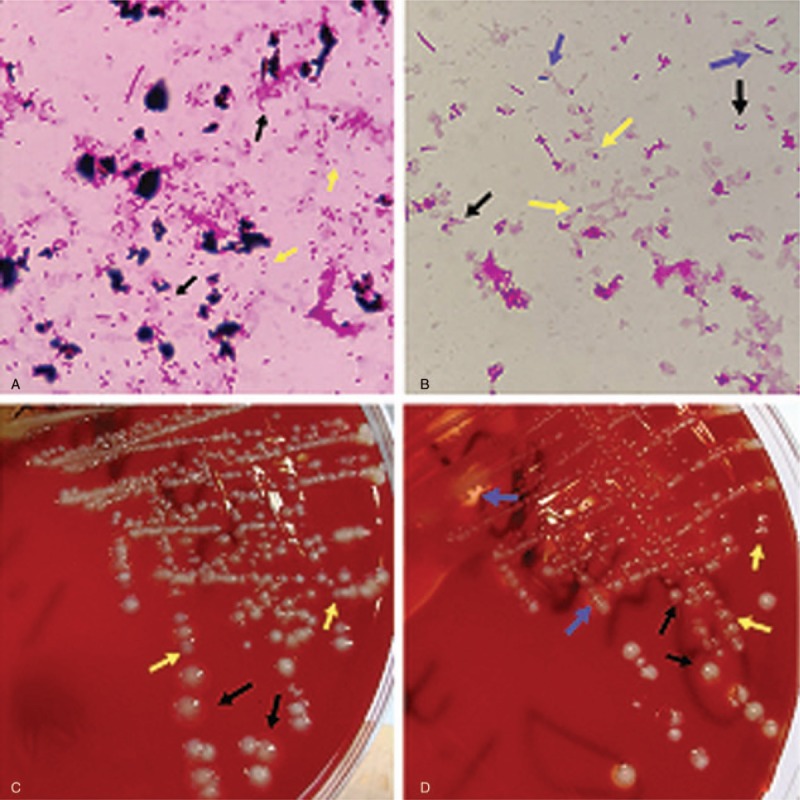
Three types of bacteria in Columbia blood agar media and Gram straining. A. Gram staining of liquid in aerobic blood culture; B. Gram staining of liquid in anaerobic blood culture; C. Two different colonies isolated in aerobic blood culture (with or without beta-hemolytic ring); D. Three different colonies were isolated from the anaerobic blood culture (double hemolytic loop colonies, beta-hemolytic loop colonies and colonies without hemolytic loop). The 3 bacteria types are represented in yellow, black, and purple arrows.

A 49-year-old male was admitted to hospital for periodic chemotherapy. The patient had a history of liver metastasis of duodenal cancer for 10 months and had undergone radical pancreaticoduodenectomy. No obvious abnormalities in the physical examination and laboratory tests were found at this point. On the third day of admission, the patient went through surgery and TACE due to chemotherapy indications without pre-operative prophylactic antibiotic administration. 20 hours following TACE the patient presented with intermittent fever (<38°C) that was regarded as post-embolization syndrome. On day 5 following TACE (December 31st, 2017), the patient's condition worsened with symptoms changing to sudden chills and high fever (up to 39.5°C). Laboratory tests revealed increased concentration of c-reactive protein (CRP) and neutrophils and increased liver function as measured by changed in bilirubin, transaminases and gamma-glutamyl transferase (GT). Critically low values of hemoglobin and hematocrit (Hct) were also detected. At this time, a blood sample was collected for 2 sets of blood cultures, anaerobic and aerobic. The patient was then treated with levofloxacin for infection, but the body temperature did not drop considerably even 19 hours after the start of antibiotic treatment. At this time, both sets of blood cultures were found positive, with Gram-positive bacilli and Gram-negative bacteria detected in the anaerobic cultures, and Gram-negative bacteria detected in the aerobic cultures (Fig. 1). Considering a possible infection of intestinal and anaerobic bacteria due to TACE, the treatment was immediately adjusted to cefoperazone sodium/sulbactam sodium in combination with ornidazole on January 1, 2018. The patient's body temperature subsequently dropped gradually after 5 hours of treatment.

**Figure 2:**
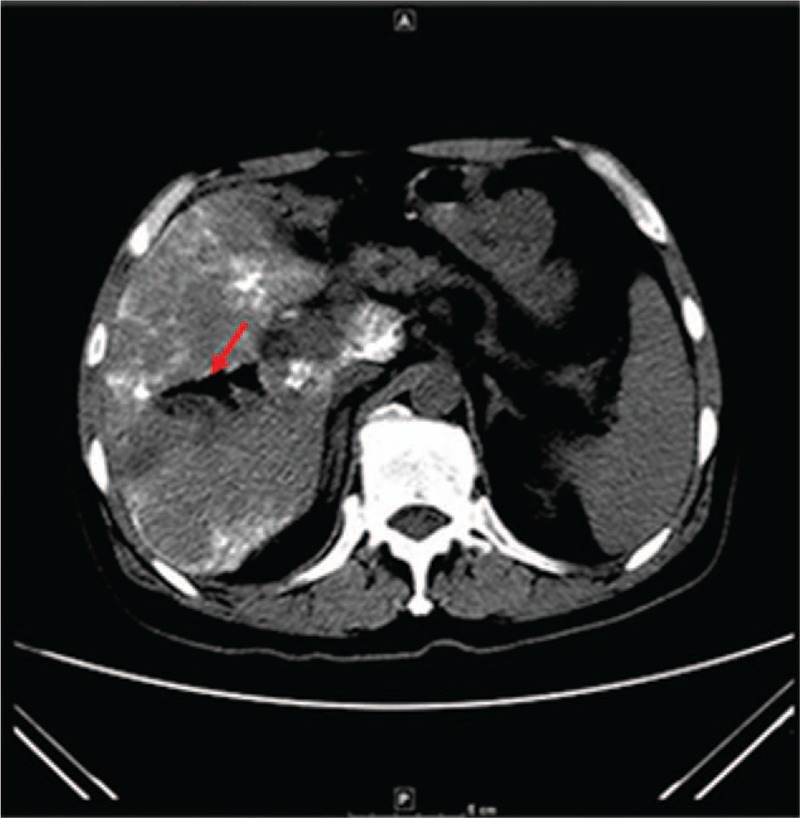
Abdominal CT scan, on January 3rd, 2018. Note: The red arrow represents accumulated gas in the liver.

On January 3, 2018, symptoms (body temperature of 39.8°C and chills) relapsed along with swelling and liver tenderness. Abdominal Computed Tomography (CT) revealed accumulated gas in the intrahepatic bile duct (Fig. 2). The patient was then diagnosed with liver abscess, and intrahepatic abscess drainage was performed immediately, followed by a malodorous maroon liquid release. Three types of bacteria were then identified *C perfringens*, *A hydrophila*, and *E coli* in both sets of anaerobic cultures. In aerobic cultures we identified two species of bacteria, *A hydrophila* and *E coli* (Fig. 1). The positive outcome of drug treatment was due to bacteria identified in the blood cultures being susceptible to the antibiotics administered. Among these, *E coli* and *A hydrophila* are sensitive to imipenem, while *E coli* is resistant to all quinolones and β-lactam antibiotics. To determine the origin of the liver abscess, the drainage fluid was tested in aerobic cultures on January 6, 2018. The findings showed the presence of *E coli,* and drug susceptibility results were consistent with these blood cultures. The clinician formulated a therapy in accordance with the drug susceptibility test and the empirical drug use for anaerobic bacteria. A comprehensive treatment plan was adopted on the basis of the combination of nitrazole and imipenem as anti-infection drugs and continuous abscess drainage. After 2 weeks of treatment, the patient was discharged, as infection was controlled and physical examination had returned to normal.

## 3 Discussion

BSI is a serious infectious disease that results in high mortality levels (in approximately 12% of diagnosed cases).^[5]^ The widespread application of invasive diagnostic techniques and the existence of a low-immunization population have increased the annual incidence of bacteremia caused by BSI. Bacteremia is typically caused by monomicrobial infections. Here, we report a case of BSI caused by 3 types of bacteria. With the exception of *E coli,* bacteremia caused by *C perfringens* and *A hydrophila* are infrequent, and infection by all 3 bacteria is even less common. The mortality rate following infection by *C perfringens* bacteremia is extremely high (ranging from 27% to 80%) and disease progression is rapid.^[2,6–8]^ The median time from onset to death from *C perfringens* bacteremia is approximately 8 to 9.7 hours.^[2,8]^ Therefore, untimely and inappropriate therapy for *C perfringens* may lead to multiple organ failure or death within a short period of time.^[9]^ Notably, BSI is usually manifested by elevated inflammatory markers, such as leukocytes, CRP, and procalcitonin. Interestingly, our patient showed a sudden increase in liver enzymes and progressive aggravation of anemia, with hemoglobin decreasing from 12.6 g/dL to 7.2 g/dL, and hematocrit decreasing from 40.7% to 21.9%, which may have been caused by *C perfringens* bacteremia. *C perfringens* bacteremia can be diagnosed by hemolyzed blood samples, elevated liver enzymes, and severe anemia.^[8]^ In our report, these nonspecific indicators did not alert the clinician's attention. The consideration of medical history in the diagnosis of *C perfringens* bacteremia is particularly important, especially in the event of gastrointestinal surgery and cancer chemotherapy, as these can be potential risk factors.^[8]^ Therefore, bacteremia may be closely associated with patients undergoing TACE. Another pathogen that is typically involved in polymicrobial bacteremia is *A hydrophila*, but it seldom plays a causal role in human BSI. However, *A hydrophila* bacteremia has been shown to have a close relationship with malignant tumors and hepatic biliary dysfunction (especially cirrhosis).^[10]^ In this case, the drug susceptibility results for *E coli* were consistent in both the drainage of liver abscess and blood cultures. Therefore, this pathogen had spread to the liver via blood circulation, thereby leading to the formation of a liver abscess. Interestingly, the 2 other bacteria types that led to BSI were not isolated from the drainage fluid, which can be attributed to the use of antibiotics and changes in bacterial culture patterns (aerobic culture only). *C perfringens*, *A hydrophila*, and *E coli* are all normal flora of the intestine and bacterial translocation is an important pathway for autoinfection. One study has revealed that various factors, such as intestinal bacterial overgrowth, intestinal mucosal barrier changes and immune dysfunction may promote intestinal bacterial translocation when the patient is critically ill.^[3]^ Accordingly, the primary disease, TACE surgery and long-term periodic chemotherapy in our patient may have caused damage to the intestinal mucosa, which was a major cause of liver abscess formation and polymicrobial BSIs.

TACE is always accompanied by a series of self-limited clinical responses known as the post-embolization syndrome, characterized by nausea, emesis, abdominal pain, loss of appetite, and daily intermittent fevers (below 39°C).^[11,12]^ Thus, when patients present with light fever, the clinician is likely to associate it with post-embolization syndrome. In this case study, it led to deterioration of the patients’ condition. Consequently, the identification of either post-embolization syndrome or bacteremia after TACE is particularly important. Prophylactic administration of antibiotics before TACE remains a controversial issue.^[4]^ Li et al^[13]^ reported the risk of complications such as infection and liver abscess in patients with a history of abdominal surgery that may increase with TACE procedures. Brown et al^[14]^ also showed that patients without an intact sphincter of Oddi are at an increased risk of abscess formation that can result in post-embolization infection and that the performance of a bowel preparation and ensuring coverage of aerobic and anaerobic organisms can reduce this risk.^[14]^ However, other studies have demonstrated that prophylactic antibiotics do not need to be administered to patients prior to TACE.^[15,16]^ Unnecessary antibiotic usage has several potential risks, such as alteration of the intestinal flora and an increase in antibiotic resistance and hospital cost. Here, we report a rare case of hepatic abscess combined with polymicrobial bacteremia in a patient with a history of radical pancreaticoduodenectomy. Infection may have originated from autogenous infection from gut pathogens.

In summary, when patients have a history of abdominal surgery and/or have low immunity, TACE may lead to secondary infections and other complications. Thus, appropriate prophylactic antibiotics may be administrated in these cases.

## Acknowledgments

We are grateful to the Department of Oncology in the Second Affiliated Hospital of Nanchang University for providing information for this case study. We thank Dr. Houqun Ying and Dr. Shubiao Zou for assistance in drafting this manuscript. We would like to thank Editage (www.editage.cn) for English language editing.

## Author contributions

**Conceptualization:** Xueyao Fang, Longhua Hu, Qiaoshi Zhong.

**Formal analysis:** Xueyao Fang.

**Investigation:** Yanhui Chen, Yanhua Liu.

**Resources:** Xiaoyan Hu.

**Supervision:** Yaping Hang.

**Visualization:** Xiuhua Xu, Nan Zhang.

**Writing – original draft:** Xueyao Fang.

**Writing – review & editing:** Longhua Hu, Qiaoshi Zhong.
